# Occupational Exposure to Welding Fumes and Associated Respiratory Morbidities among arc Welders in Ikenne, Nigeria

**DOI:** 10.4314/ejhs.v33i2.23

**Published:** 2023-03

**Authors:** Bamidele Emmanuel Fikayo, Okebalama Victor Chimezie, Sodeinde Kolawole John, Ogunkoya John Omotola, Ivy Chizurum Mbon, Peace Chioma Eleonu, Kelechi Walter Ndinne, Taiwo Adesola Atinuke

**Affiliations:** 1 Department of Community Medicine, Babcock University Teaching Hospital, Ilishan-Remo, Ogun State, Nigeria; 2 Department of Anatomical Pathology and Forensic Medicine, Babcock University Teaching Hospital, Ilishan-Remo, Ogun State, Nigeria; 3 Department of Internal Medicine, Babcock University Teaching Hospital, Ilishan-Remo, Ogun State, Nigeria; 4 Department of Surgery, Babcock University Teaching Hospital, Ilishan-Remo, Ogun State, Nigeria; 5 National Health Insurance Clinic, Department of Family Medicine, the University of Portsharcourt Teaching Hospital, Rivers State, Nigeria; 6 Faydat Athqab Primary Healthcare Center, Hail, Saudi Arabia; 7 Benjamin S. Carson (Snr) College of Health and Medical Sciences, Babcock University, Ilishan-Remo, Ogun State, Nigeria

**Keywords:** Lung function test, Welders, Fume exposure

## Abstract

**Background:**

Few studies have been conducted on the respiratory morbidities of welders in Nigeria, and further research is needed to determine the extent of fume exposure and the implicated metals at workplaces. This study was done to determine whether welding gases are associated with respiratory illness among welders in Ikenne Local Government Area, Ogun State, Nigeria.

**Methods:**

A cross-sectional study comparing 142 welders and 142 controls was carried out. Lung function testing and clinical assessments were done. The Chi-square test & the independent T-tests were used to test for the association between socio-demographic characteristics and respiratory symptoms & the association between differences in means of lung function parameters among welders and controls respectively.

**Results:**

Chromium, nickel, manganese, carbon monoxide, and nitrogen dioxide levels were higher than the recommended values. Forced expiratory volume in the first second (FEV1) was significantly lower in welders (2.62±0.7) than in controls (2.81±0.7) (t=2.148, p<0.05). FEV1/FVC was significantly lower among welders (75±13.7) compared to controls (80.7±8.0). (t=4.165, p<0.001).

**Conclusion:**

The study showed that the welders presented with more respiratory morbidities than the controls, this may be a result of exposure to high levels of welding fumes beyond the recommended values for prolonged periods without using personal protective equipment, which results in significant morbidities. There should be enforcement of basic workplace safety standards by ensuring that the use of personal protective equipment (PPE) is enforced and the construction of workshops that are well-ventilated through the welders' association and relevant law enforcement agencies.

## Introduction

Welding is defined as a process of joining metals together to form a union by heating to a suitable temperature with or without the use of filler metal to form a permanent (homogenous) bond (1, 2). According to the national institute of occupational safety and health (NIOSH), there are about two (2) million welders globally (3). There are over eighty (80) different types of welding processes in commercial use (4). The route of exposure to welding fumes in human beings is mainly through the lungs while the other routes include the skin, ear, and eyes (2).

Epidemiological studies have shown that a large number of welders experience some type of respiratory illness. Respiratory effects seen in full-time welders include bronchitis, siderosis, asthma, and a possible increase in lung cancer. Pulmonary infections are increased in terms of severity, duration, and frequency among welders (5).

Several pieces of research have also reported an increased frequency of chronic bronchitis and the prevalence of respiratory symptoms among welders with risk ratios varying between 1.2 and 3.1 (6–8). This finding was further supported by a survey of full-time welders, where it was reported that an increase in the prevalence of symptoms of chronic bronchitis is the most frequent problem that is associated with respiratory health (9, 10).

The health hazards of welding fumes and gases are determined by the type of welding process, base metal, and filler metals used, welding rod composition, work environment, protective measures used, welder working practices, and length of time of exposure (11). However, exposure may cause short-term or long-term health effects. (12, 13)

Short-term effects include irritation of the eyes, nose, chest, and respiratory tract, which can manifest as cough, wheezing, difficulty in breathing, bronchitis, pulmonary oedema, and metal fume fever, which are a result of exposure to metal fumes like zinc, magnesium, copper, and copper oxide whereas the long-term effects include cancer of the lungs and urinary tract, chronic bronchitis, pneumonia, asthma, and reduced lung function (12, 13). Studies in the past have shown a small decline in pulmonary function among welders (14, 15). Also, the long-term effect is not very clear, but there is a high probability that these declines could result in the development of respiratory impairments. There has been an increase in the rising cases of welding-related illnesses in Nigeria (16). A survey done in Benin City, Nigeria reported a prevalence of 96.4% for work-related health complaints among welders, and 43% of them also suffered from metal fume fever (16). A study done among eight welding sites found an acute decrease in FeV_1_ relative to work more among the welders than non-welders (17). In Nigeria, studies on arc welding are few. While most of these studies focused more on respiratory symptoms and eye problems, others focused on the pulmonary functions of welders and the use of personal protective equipment, (4) but the level of the fume exposure and metals implicated at their workplaces remain to be explored. Besides, the previous studies did not estimate the level of exposure of welders to metal fume at their workplace. Therefore, this study aims to assess the level of welding fumes, respiratory symptoms related to welding fumes exposure, and pulmonary function of welders in Ikenne local government area, Ogun State, Southwest, Nigeria.

## Materials and Methods

The study was a comparative cross-sectional study, carried out in Ikenne Local Government in Ogun State, Southwest Nigeria. The study population consisted of male arc welders working in towns located within Ikenne local government area, who were exposed to welding fume and control were male security officers working at Babcock University, Ilisan-Remo, Ikenne LGA. The sample size was calculated using the formula for the comparison of the means of two (2) independent groups (18). The sample size was calculated using the formula for the comparison of means of two (2) independent groups



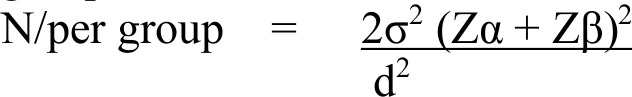



Where N/per group: minimum sample size per group

Zα = Standard normal deviation of α at 95% C.I =1.96

Zβ = Standard normal deviation of β at 80% C.I = 0.84

d = mean difference of FEV_1_ between the 2 groups (19)

χ = Standard deviation of FEV_1_ = 0.57



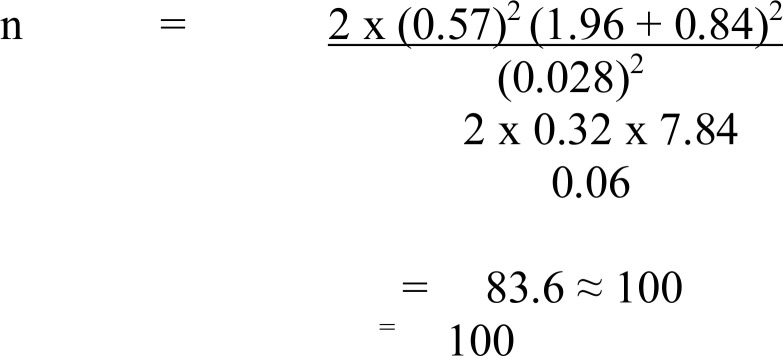



Considering the non-response factor which is 10% of the sample size:

= 100/0.9

= 111.1 ≈111 per group

The sample size was 111 for each arm of the study. The minimum sample size estimation was 100, correcting for a possible non-response rate of 10%, and the final calculation was 111. The sample size was 111 for each arm of the study. One hundred and forty-two welding workers were recruited for the study (n=142). The lists of the welders were obtained from the chairman of the welders' association in each town (Ilisan, Ikenne, Iperu, Ogere, and Irolu). The welders were sampled proportionately from each town based on the number of registered members (202), and a computer-generated simple random scheme was used to pick the subjects. (60 subjects from Iperu, 30 subjects from Ilisan, 20 subjects from Ikenne, 18 subjects from Ogere, and 14 subjects from Irolu).

For the control group, 142 male subjects were selected by a computer-generated simple random scheme from the sampling frame of 200 security officers working at Babcock University. The security officers reside in different towns within Ikenne LGA, and security officers leaving beside a welder's workshops were excluded from the study. All the welders and security officers that were recruited into the study underwent a pulmonary function test.

A validated questionnaire on respiratory symptoms approved by the Medical Research Council's Committee on Environmental and Occupational Health (20) and a proforma to obtain respondents' biodata, weight, height, blood pressure, and spirometric measurement were adapted to the study setting. It consisted of 3 sections, viz: Section A contained questions on socio-demographic characteristics, section B contained questions on the use of personal protective equipment with 4 items, while section C contained questions on respiratory and other health-related symptoms associated with welding with 25 items. Pulmonary function tests were conducted using Spirolab III(R) (MIR Italy). Spirometry was carried out following the recommendations of the American thoracic society (21). The test was performed within a fixed period (9 am to 12 pm) local time to minimize diurnal variations (22).

Spirometry was performed with participants sitting in front of the apparatus without using a nose clip and with all tight clothing loosened. At least three (3) measurements were required from each welder for analysis. The best measurement was selected to obtain the forced vital capacity (FVC) and the forced expiratory volume in one second (FEV_1_). Outcome variable: The values of FVC, FEV_1_, FEV_1_ / FVC were examined and categorized as either normal result, obstructive defect, restrictive pattern, or mixed pattern.

**Table:** 

FVC Diagnosis	FEV_1_/FVC
≥LLN	≥LLN or ≥70%
normal	
>LLN	<LLN or <70%
obstructive defect	
<LLN	≥LLN or ≥70%
restrictive pattern	
< LLN	<LLN or <70%
mixed pattern	

LLN: the lower limit of normal: defined as below the fifth percentile of spirometry data obtained from The Third National Health and Nutrition Examination Survey (NHANES III) (23).

Half of the total workshop areas in Ikenne LGA (n=72) were selected by a computer-generated simple random scheme per town and were assessed for the particulate matter and metallic constituents of the welding fumes. The analysis of the particulate matter was done according to the guidelines (21). The air monitoring was done in thirty-six selected welders' workshops. The equipment used for sampling included a cellulose ester filter, cassettes, and an air sampling pump. SKC-224-52 MTX air sampling pump made in the UK and cellulose ester filter with 0.8µm in pore size and 37mm in diameter was used.

**Procedure**: The air sampling pump was calibrated before use, while the cellulose filter was placed in the cassette before inserting it into the pump. The preliminary weight of the filters was recorded by the technician before placing them in the cassette. The filter was analyzed to determine the concentrations of total particulate fume by subtracting the preliminary weight from the secondary weight, while metallic constituents of the fume were obtained through analysis of the filter, using the Agilent 7900 ICP-MS (Inductively coupled plasma-Mass Spectrometry) which was done in the biochemistry laboratory in Redeemers University. The results were compared with controls and Standard workplace exposure limits.

**Data analysis**: The questionnaires were cross-checked, cleaned, and analysis was done using the IBM SPSS version 20 (SPSS 20). The level of significance was set at 5% (0.05). Data analysis included descriptive statistics for the socio-demographic characteristics, knowledge, and use of personal protective equipment. Respiratory and other health symptoms were analyzed using frequency tables. Means were used to summarize the age, weight, and height. The Chi-square test was used to test for an association between selected socio-demographic characteristics and respiratory symptoms.

The independent T-test was used to test the difference in means of lung function parameters among welders and security officers.

**Ethical considerations**: Ethical clearance was obtained from Babcock University Health Research and Ethics Committee (BUHREC437/19). Permission was also obtained from both welders' and security officers' associations. Written informed consent was obtained from each of the respondents. Strict ethical standards and procedures were adhered to and the anonymity of the participants was ensured by not having any identification on the data collection tool so that information could not be traced back to the individual. The data was stored on computers that had passwords. All the participants were informed about the study and its importance. Any participants who wished to withdraw from the study were allowed to do so.

## Results

**The response rate among welders was 100% while it was 95.8% among controls**: Table 1 shows the socio-demographic characteristics of respondents. About one-quarter of the welders (24.6%) and about two-fifth of security officers (39.7%) were within the age group of 30–39 years. The difference in the mean age of welders (36.8%) and security officers (36.7) was not statistically significant (t-0.094, p=0.93). About two-fifth of the welders (38.7%) were married as against 50% among security officers. Only 8 welders (5.6%) had tertiary education as the highest academic attainment as compared with two-fifth (40.4%) among security officers. Most of the welders (79.6%) and less than three-fifths of the security officers (57.4%) had a minimum of secondary education. Less than a fifth of the welders (14.8%) currently smoked cigarettes as against none among security officers.

**Table 1:** Socio-demographic characteristics of respondents

Variable	Welder n=142 (%)	Control n=136 (%)	Test statistics
**Age (Years):**			
<20	24 (16.9)	1 (0.7)	
21–29	22 (15.5)	33 (24.3)	
30–39	35 (24.6)	54 (39.7)	
41–49	30 (21.2)	34 (25.0)	
50–59	25(17.6)	10 (7.4)	
≥60	6 (4.2)	4 (2.9)	
**Mean**	**36.8±13.9**	**36.7±9.4**	**t-0.094, p=0.93**
**Marital Status:**			
Single	5 (3.5)	62 (45.6)	
Married	55 (38.7)	68 (50.0)	
Separated	81 (57.0)	3 (2.2)	
Widower	1 (0.7)	2 (1.5)	
Divorced	0 (0)	1 (0.7)	χ^2^=123.6, p< 0.001
**Educational Status:**			
Primary	21 (14.8)	2 (1.5)	
Secondary	113 (79.6)	78 (57.4)	
Tertiary	8 (5.6)	55 (40.4)	
Others	0 (0.0)	1 (0.7)	χ^2^=58.1, p < 0.001
**Cigarette Smoking:**			
Currently Smoked	21 (14.8)	0 (0.0)	
Never smoked	113 (79.6)	128 (94.1)	
Previously smoked	8 (5.6)	8 (5.9)	χ^2^=21.8, p< 0.001

Table 2 shows the occupational history of welders. About two-fifth (40.8%) of the welders had less than 10 years of work experience, while only a few (4.2%) had worked as professional welders for 40 years or more. About half (50.7%) of them worked six days a week and many of them (54.9%) worked for more than eight hours daily.

**Table 2:** Occupational history among welders (n=142)

Variable	Frequency (%)
**Work experience (in years)**	
<10	58 (40.8)
10–19	28 (19.7)
20–29	24 (16.9)
30–39	26 (18.3)
≥ 40	06 (4.2)
**No of days of working per week**	
5	10 (7.0)
6	72 (50.7)
7	60 (42.3)
**Duration of exposure(per day)**	
<8	64 (45.1)
>8	78 (54.9)

Table 3 shows the use of personal protective equipment among welders. Six (4.4%) of them use a helmet always when working, four (2.8%) use it occasionally, and three (2.1%) rarely use a helmet when working. Fourteen (9.8%) of them make use of a respirator always while three (2.1%) rarely use it when welding. Ten (7.0%) of them use safety boots always, four (2.8%) use them occasionally, and six (4.2%) rarely do. One hundred and thirteen (79.6%) of welders in our study use eye goggles always, 15 (10.6%), and five (3.5%) use them occasionally and rarely, respectively. Twelve welders (8.4%) use an apron always, while four (2.8%) use it both occasionally and rarely respectively. Only one (0.7%) of the welders use earplugs always and rarely and 13 (49.1%) use welding gloves always, while nine (6.3%) and eleven (7.7%) of welders use them occasionally and rarely, respectively.

**Table 3:** Use of personal protective equipment among welders (n=142)

Variable	Always	Occasionally	Rarely	Never
Use of Helmet	6 (4.2%)	4 (2.8%)	3 (2.1%)	129 (90.8%)
Respirator	14 (9.8%)	0 (0.0%)	3 (2.1%)	125 (88.0%)
Safety booth	10 (7.0%)	4 (2.8%)	6 (4.2%)	122 (85.9%)
Eye goggle	113 (79.6%)	15 (10.6%)	5 (3.5%)	9 (6.3%)
Apron	12 (8.4%)	4 (2.8%)	4 (2.8%)	122 (85.9%)
Ear muff	1 (0.7%)	0 (0.0%)	1 (0.7%)	140 (98.6%)
Welding gloves	13(9.1%)	9(6.3%)	11(7.7%)	109(76.7%)

Table 4 shows the prevalence of respiratory symptoms among respondents. Thirty-two (22.5%) of the welders experienced cough during the day, while twenty-three (16.9%) security officers coughed during the day (p=0.24). About one-fifth (21.1%) of welders produced sputum during the day compared to less than one-tenth of the security officers (p<0.001). Ten (7.0%) of the welders wheezed during the day compared to nine (6.6%) security officers (p=0.89). Fifty welders (36.6%) had catarrh compared to 5.9% of the security officers who had catarrh (p<0.001). Only sputum production and catarrh among the welders showed significant differences while other symptoms were not compared to the controls

**Table 4:** Respiratory symptoms among welders and controls

Variable	Welders n=142		Control n=136		Test statistics
	
	Yes (%)	No (%)	Yes (%)	No (%)	
Cough during the day	32 (22.5)	110 (77.5)	23 (16.9)	113 (83.1)	χ^2^=1.38, P=0.24
Cough at night	12 (8.5%)	130 (91.5%)	20 (14.7%)	116 (85.3%)	χ^2^=2.66,p=0.10
Cough for at least 3 consecutive months for the last year consecutive years	8 (5.6)	134 (94.4)	4 (2.9%)	132 (97.1)	χ^2^=1.22,p=0.27
The cough gets worse when working	7 (4.9)	135 (95.1)	3 (2.2%)	133 (97.8%)	χ^2^=1.49,p=0.22
Cough improves when not working	17 (12.0)	125 (88.0)	8 (95.9%)	128 (94.1%)	χ^2^=3.15,p=0.08
Sputum during the day	30 (21.1%)	112 (78.9%)	10 (7.4%)	126 (92.6%)	χ^2^=10.7,p < 0.001
Sputum at night	6 (4.2%)	136 (95.8%)	6 (4.4%)	130 (95.6%)	χ^2^=0.006,p=0.94
Sputum for at least 3/12	5 (3.5%)	137 (96.5%)	2 (1.5%)	134 (98.5%)	χ^2^=1.19,p=0.28
Sputum gets worse when working	5 (3.5%)	137 (96.5%)	4 (2.9%)	132 (97.1%)	χ^2^=0.08,p=0.79
Sputum gets worse when not working	4 (2.8%)	138 (97.2%)	3 (2.2%)	133 (97.8%)	χ^2^=0.11,p=0.75
Chronic Bronchitis	13(9.1%)	129(90.0%)	6(6.6%)	130(93.4%)	
Wheezing during the day	10 (7.0%)	132 (93.0%)	9 (6.6%)	127 (93.4%)	χ^2^=0.02,p=0.89
Wheeze at night	4 (2.8%)	138 (97.2%)	4 (2.9%)	132 (97.1%)	χ^2^<0.004, p=0.95
Wheeze for at least 3/12 in the last 12 months	5 (3.5%)	137 (96.5%)	6 (4.4%)	130 (95.6%)	χ^2^=1.15,p=0.70
Wheeze gets worse on working	6 (4.2%)	136 (95.8%)	3 (2.2%)	133 (97.8%)	χ^2^0.90,p=0.06
Dyspnea when walking on level ground	8 (5.6%)	134 (94.4%)	3 (2.2%)	133 (97.8%)	χ^2^=2.15,p=0.14
Dyspnea when going uphill	11 (7.7%)	131 (92.3%)	14 (10.3%)	122 (89.7%)	χ^2^=0.55,p=0.46
Dyspnea when not working	2 (1.4%)	140 (98.6%)	0 (0.0%)	136 (100%)	χ^2^=1.93,p=0.17
Dyspnea gets worse when working	2 (1.4%)	140 (98.6%)	3 (2.2%)	133(97.8%)	χ^2^=0.25,p=0.62
Catarrh	52 (36.6%)	90 (63.4%)	8(5.9%)	128 (94.1%)	χ^2^=38.8p <0.001
Asthma	3 (2.1%)	139 (97.9%)	4 (2.9%)	132 (97.1%)	χ^2^=1.19,p=0.66
Asthma gets worse when working	6 (4.2%)	136 (95.8%)	5 (3.7%)	131 (96.3%)	χ^2^=0.05,p=0.81

Table 5 shows the lung function parameters and pattern of lung function tests among the respondents. The forced expiratory volume in the first second (FEV1) was significantly lower in welders (2.62±0.7) than in controls (2.81±0.7) (t=2.148, p=0.05), forced vital capacity (FVC) was slightly higher in welders (3.50±0.9) than in controls (3.44±0.99), however not statistically significant (t-581, p=0.56) while FEV1/FVC was significantly lower among welders (75.07±13.7) than in controls (80.7±8.0) (t: 4.165, p<0.001). Over a hundred security officers (92.6%) had normal lung function as compared to ninety-seven welders (68.3%). Nearly one-third of the welders (31.0%) had obstructive lung function compared to ten (7.4%) of the security officers, while only one welder had restrictive lung function (0.7%) as against none among the security officers.

**Table 5:** Lung Function Values and pattern of lung function tests of Respondents

Variable	Welder n=142	Security n=136	Test statistics
FEV_1_(l/min)SD	2.62±0.7	2.81±0.7	t:2.148,p=0.05
FVC (l) SD	3.50±0.9	3.44±0.9	t:-0.581,p=0.56
FEV1/FVC (%)	75.07±13.7	80.70±8.0	t:4.165,p<0.001
Normal result	97(68.3)	126(92.6)	
Obstructive pattern	44(31.0)	10(7.4)	χ^2^=26.1,p<0.001
Restrictive pattern	1(0.7)	0	
Mixed pattern	0	0	

Table 6 shows that some particulate concentrations were high compared to controls. The air chromium and nickel concentration in welders' workplaces were 2 times higher than in controls. Manganese was four times higher, while Iron, carbon monoxide, zinc, and copper were slightly higher in welders' workplaces compared to controls.

**Table 6:** Eight-hour average particulate concentration measurement in thirty-six selected workshops

Metallic Particles (mg.m^-3^	Cr	Ni	Co	Fe	Mn	Cu	Zn	Al
Iperu welders	10.80	12.53	46.50	5.63	42.75	1.50	4.28	3.86
Ilisan welders	12.56	12.94	49.88	5.25	43.88	2.25	4.45	3.98
Ikenne welders	10.39	11.18	48.00	4.50	42.00	1.13	4.58	3.30
Ogere welders	11.18	10.80	47.25	5.25	43.50	2.25	4.35	2.85
Irolu welders	10.60	11.18	47.60	5.20	42.00	1.13	4.58	3.60
Welders average	11.10	11.73	47.85	5.17	42.83	1.65	4.45	3.52
EH/40ss/2005	0.50	0.10	23.00	5.00	0.20	1.00	1.00	10.0

## Discussion

The study aimed to identify welding fumes as a probable cause of morbidity among welders in Ikenne Local Government Area, Ogun State, Nigeria. The response rate among welders was 100% while it was 95.8% among controls.

The level of welding fumes and gas produced during welding was markedly high. The values were higher than the recommended values by EH40/2005 workplace exposure limits Exposure to such a high level of metals and gases can lead to irritation of the respiratory system, chest pain, difficulty in breathing, increase risk of lung cancer, etc., For welders to be fit for work and be productive, there is a need to educate them on the risk of exposure to such high level of welding fumes and how to mitigate its impact by wearing personal protective equipment like a welding respirator, working in a ventilated environment and need to go for the periodic medical examination to reduce the incidence of serious pulmonary diseases.

The welders in this study were exposed mainly to chromium, nickel, and manganese. This is similar to a study done among welders in Lodz, Poland that reported that manganese and chromium exceeded the threshold limit values (24). Exposure of welders to these high levels of fumes may irritate the eyes, nose, and throat, increasing the risk of lung cancer and metal fume fevers (12). The high level of welding fumes in this study is consistent with the findings of other studies (25–28). A study of exposure to welding fume among welders in Beijing observed that the levels of manganese and iron in welders were 4.3-fold and 1.9-fold higher than those of controls (25). Another study among construction welders in Korea reported that construction welders experience a risk of exposure to welding hazards at a level exceeding the exposure limits (26). Similar studies in Jeddah, Saudi Arabia showed that welders in most factories were exposed to welding fume concentrations above the Saudi Arabian standard organization (SASO) limit value (28). The studies are similar to the present study which documents that respiratory mask was rarely worn by the participants (25, 28). This corroborates with the present study which showed that the majority of the welders were not using a respirator when working.

In the present study, exposure to a high level of fume may be due to poor use of personal protective equipment (PPE). As reported in this study, only 9.8% of the welders regularly used a respirator when welding. Also, welding in a poorly well-ventilated workshop may increase exposure to a high level of fumes and gases. This shows that the welders worked in conditions that are harmful to their health due to exposure to metals and gases that exceeded recommended limits.

This study showed that FEV_1_ and FEV_1_/FVC were significantly lower among welders compared to the controls. About a third of the welder had an obstructive pattern of pulmonary function impairment. This may be because high exposure on daily basis to welding fumes increases the opportunity to develop these patterns of pulmonary function impairment among welders more than the controls. Also, this pattern of lung function impairment is more common among welders that worked for a long duration at work, as shown in this study, the majority of the welders worked throughout the week for more than 8 hours duration, which shows that working for a long period predisposes a welder to abnormal lung function impairment. This finding is similar to a study of lung function test of welders in Zaria, Nigeria which reported that a decrease in FVC, FEV_1_/FVC, and peak expiratory flow rate (PEFR) was attributed to an increase in years of exposure to welding fumes (29). Another study that assessed the lung function among welders in south Tehran showed that FEV_1_ and PEFR were significantly reduced which was attributed to exposure to various harmful welding materials (30). Like our study, several other studies have also shown a relationship between a reduction in lung function tests and an increased duration of exposure to welding fumes (6–8).

The prevalence of respiratory symptoms was higher among welders compared to controls. The major presenting symptoms were cough, sputum, and catarrh. The higher prevalence of respiratory symptoms among welders may be due to exposure to welding fumes. Daily exposure to welding fumes like magnesium, and copper may irritate the respiratory tract and result in cough, wheezing, and bronchitis (6, 8). In this study, the welder rarely used any protective equipment such as a respirator, or helmet and was exposed to welding fumes this may have also contributed to the increase in the prevalence of respiratory symptoms.

The results were in line with the results of several studies done in the past. A study done in Ahvaz, Iran reported that respiratory symptoms are more prevalent among welders than non-welders (31). Another study was conducted on the respiratory health of a population of welders in Saudi Aramco. Reported a higher prevalence of respiratory symptoms among welders than the control (6). A population-based study on welding exposures to work and respiratory symptoms in Europe found a higher level of chronic bronchitis among welders than in control, the study concluded that there is an association between welding of work with chronic bronchitis (8).

In conclusion, the level of welding fumes produced during welding was high. The values were higher than the recommended values by EH40/2005 workplace exposure limits. Chromium, nickel, and manganese levels were higher than the recommended values Duration of exposure to high levels of welding fumes and non-usage of personal protective equipment are some of the risk factors among welders. Pulmonary function tests among welders showed a significant decrease compared to controls, with most of the welders presenting with obstructive abnormal lung function patterns. The prevalence of respiratory symptoms among welders was significantly higher compared with controls. Therefore, the welders should imbibe the culture of using respiratory protection equipment to protect themselves from welding fumes and also reduce the period of exposure to welding fumes on both a daily and weekly basis e.g., they should work for less than 8 hours a day for 5 days a week. They should be enlightened on the health effects of welding fumes and the methods to minimize exposure to the fumes.

The study used aerial sampling to measure the fume exposure due to lack of finance and short duration, personal sampling would be better to assess the welders individually. Also, the smoking status of the study participants was not accounted for in this study.
